# Supplementary material for: Bioinspired Synthesis of Magnetic Nanoparticles Based on Iron Oxides Using Orange Waste and Their Application as Photo-Activated Antibacterial Agents
Source: Int J Mol Sci. 2023 Mar 1;24(5):4770. doi: 10.3390/ijms24054770 (PMC10002579; doi:10.3390/ijms24054770)
Supplement: Supplementary file 1 [file ijms-24-04770-s001.zip › ijms-2144178-supplementary.pdf]

Supplementary Material

Bioinspired Synthesis of Magnetic Nanoparticles Based on Iron Oxides Using Orange Waste and Their Application as Photo-Activated Antibacterial Agents

David Giancarlo García [1], Cristina Garzón-Romero [2], Mateo Alejandro Salazar [2], Karina J. Lagos [1], Kleber Orlando Campaña [1], Alexis Debut [3], Karla Vizuete [3], Miryan Rosita Rivera [2], Dario Niebieskikwiat [4], Maria J. Benitez [5] and María Paulina Romero [1,*]

[1] Department of Materials, Escuela Politécnica Nacional (EPN), Quito 170525, Ecuador
[2] Laboratorio de Investigación en Citogenética y Biomoléculas de Anfibios (LICBA), Centro de Investigación para la Salud en América Latina (CISeAL), Facultad de Ciencias Exactas y Naturales, Pontificia Universidad Católica del Ecuador (PUCE), Quito 170143, Ecuador
[3] Centro de Nanociencia y Nanotecnología, Universidad de Las Fuerzas Armadas ESPE, Sangolquí 171103, Ecuador
[4] Departamento de Física, Colegio de Ciencias e Ingenierías, Universidad San Francisco de Quito, Quito 170901, Ecuador
[5] Departamento de Física, Escuela Politécnica Nacional (EPN), Quito 170525, Ecuador
* Correspondence: maria.romerom@epn.edu.ec

Supplementary Figures

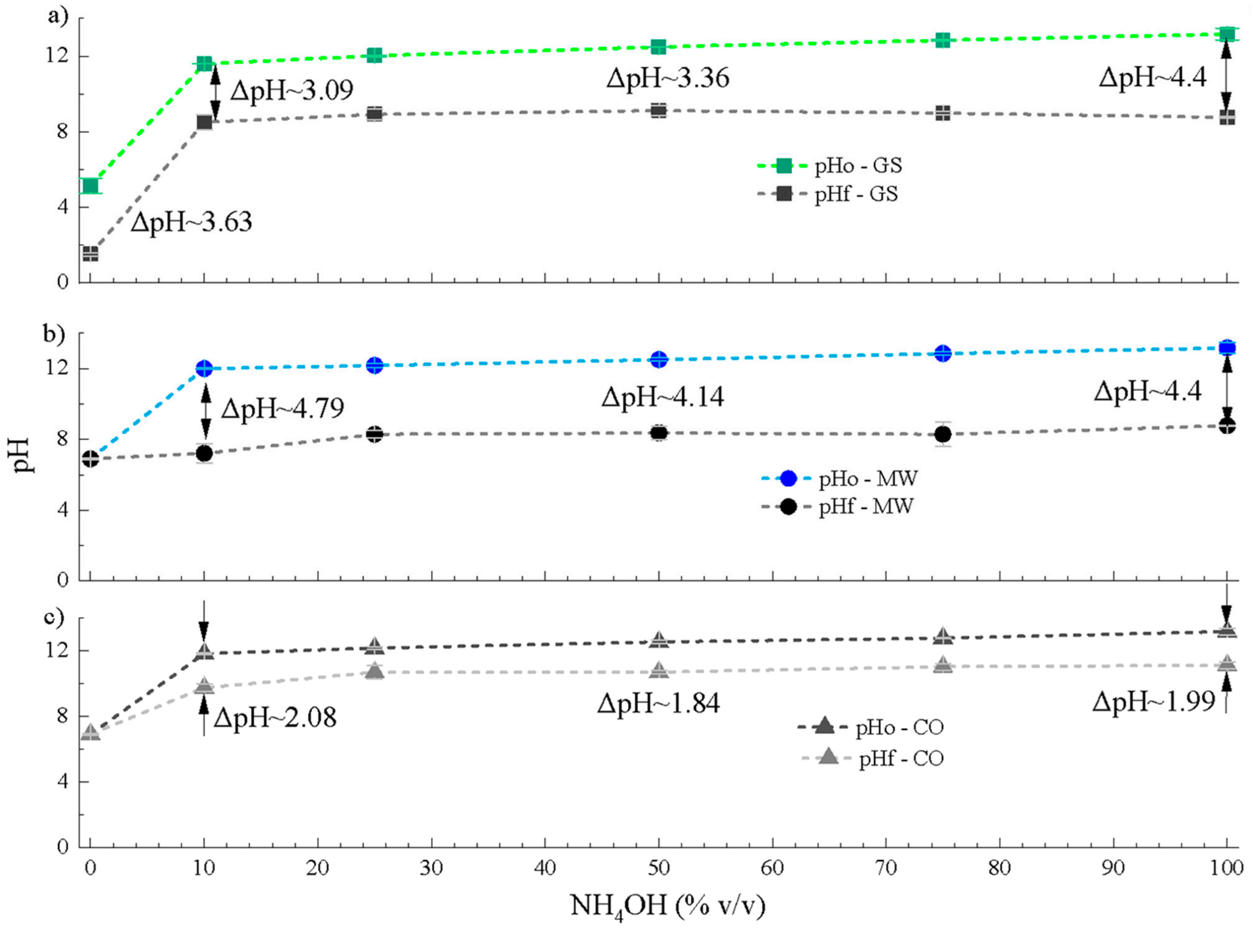

Figure S1. Initial and final pH measured in: a) green synthesis, b) microwave synthesis, and c) coprecipitation synthesis.

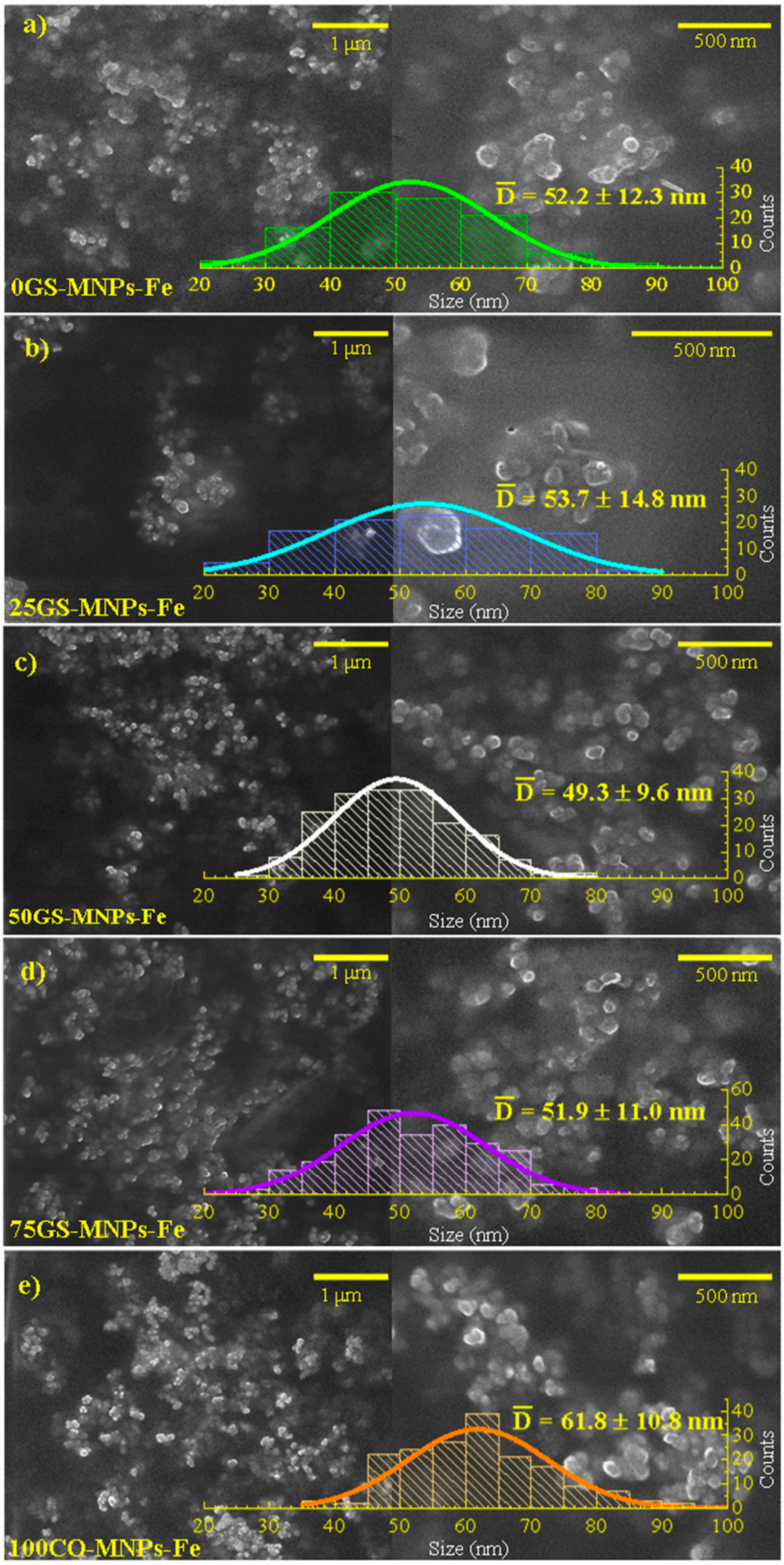

Figure S2. SEM images of the samples a) 0GS-MNPs-Fe, b) 25GS-MNPs-Fe, c) 50GS-MNPs-Fe, d) 75GS-MNPs-Fe and e) 100CO-MNPs-Fe. Filters used: Smooth, Gaussian blur (sigma radius: 1.0) and Median (Radius 0.8 pixels).

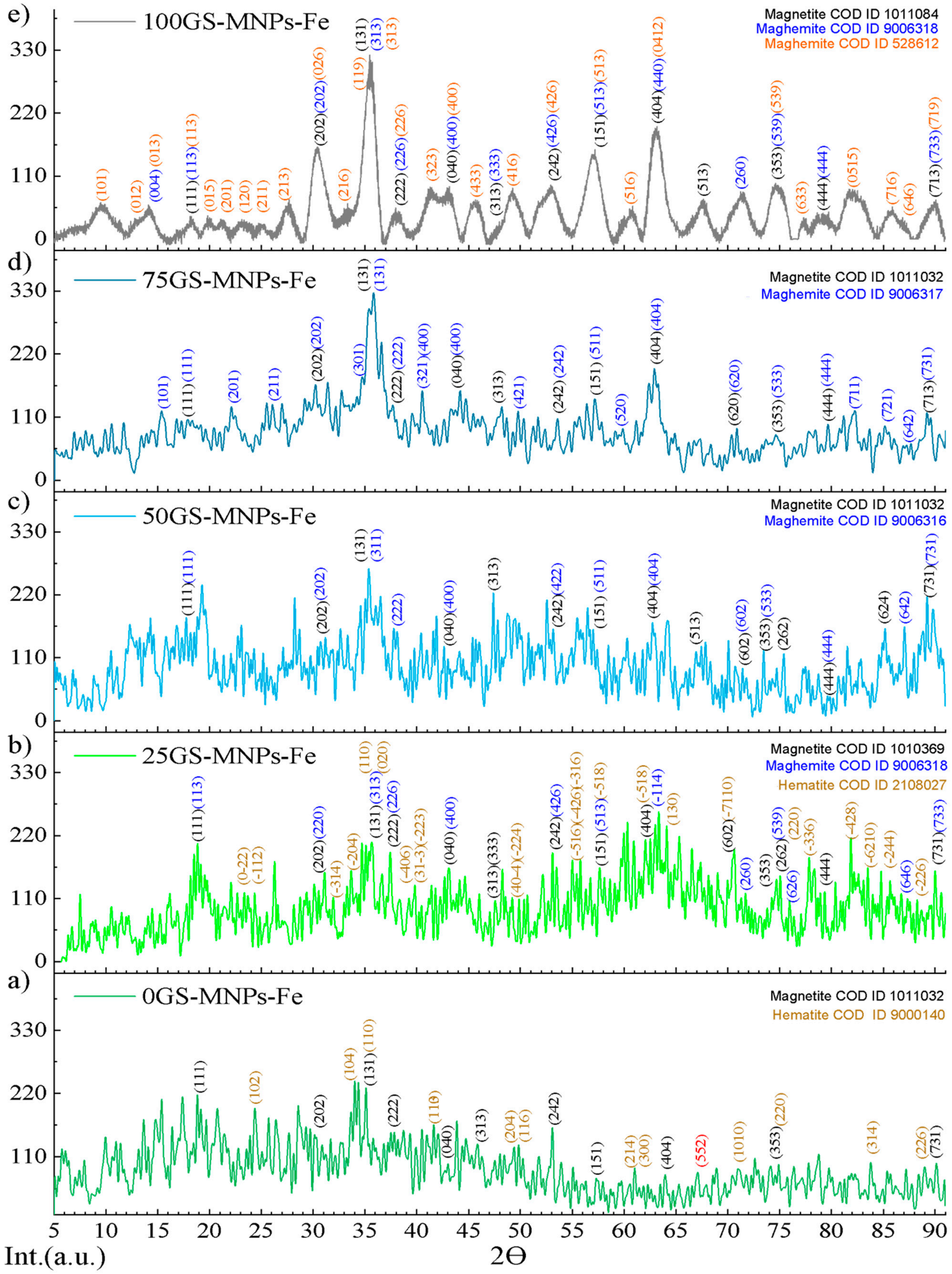

Figure S3a. Indexed XRD patterns of the samples; a) 0GS-MNPs-Fe, b) 25GS-MNPs-Fe, c) 50GS-MNPs-Fe, d) 75GS-MNPs-Fe and e) 100GS-MNPs-Fe.

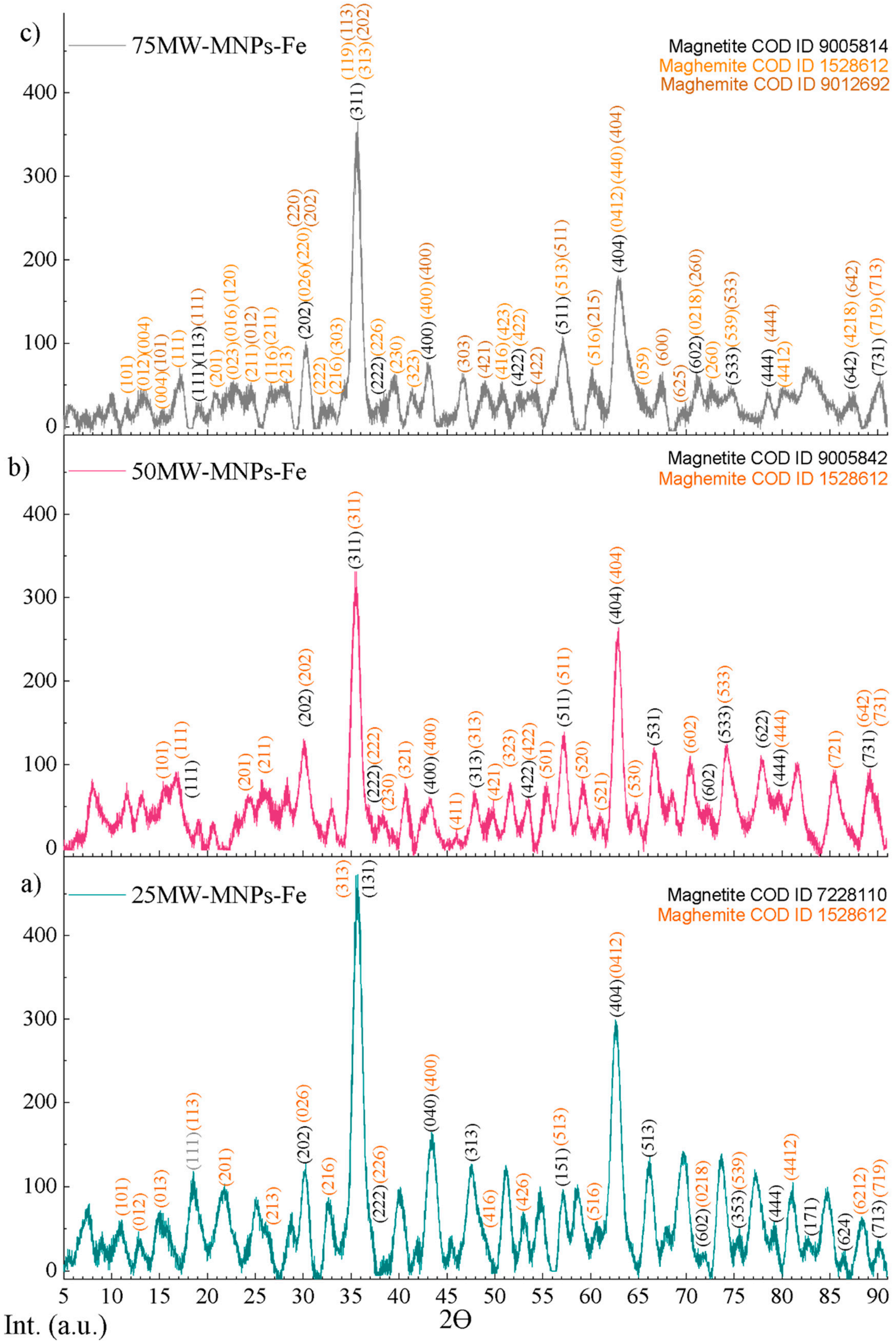

Figure S3b. Indexed XRD patterns of the samples: a) 25MW-MNPs-Fe, b) 50MW-MNPs-Fe and c) 75MW-MNPs-Fe.

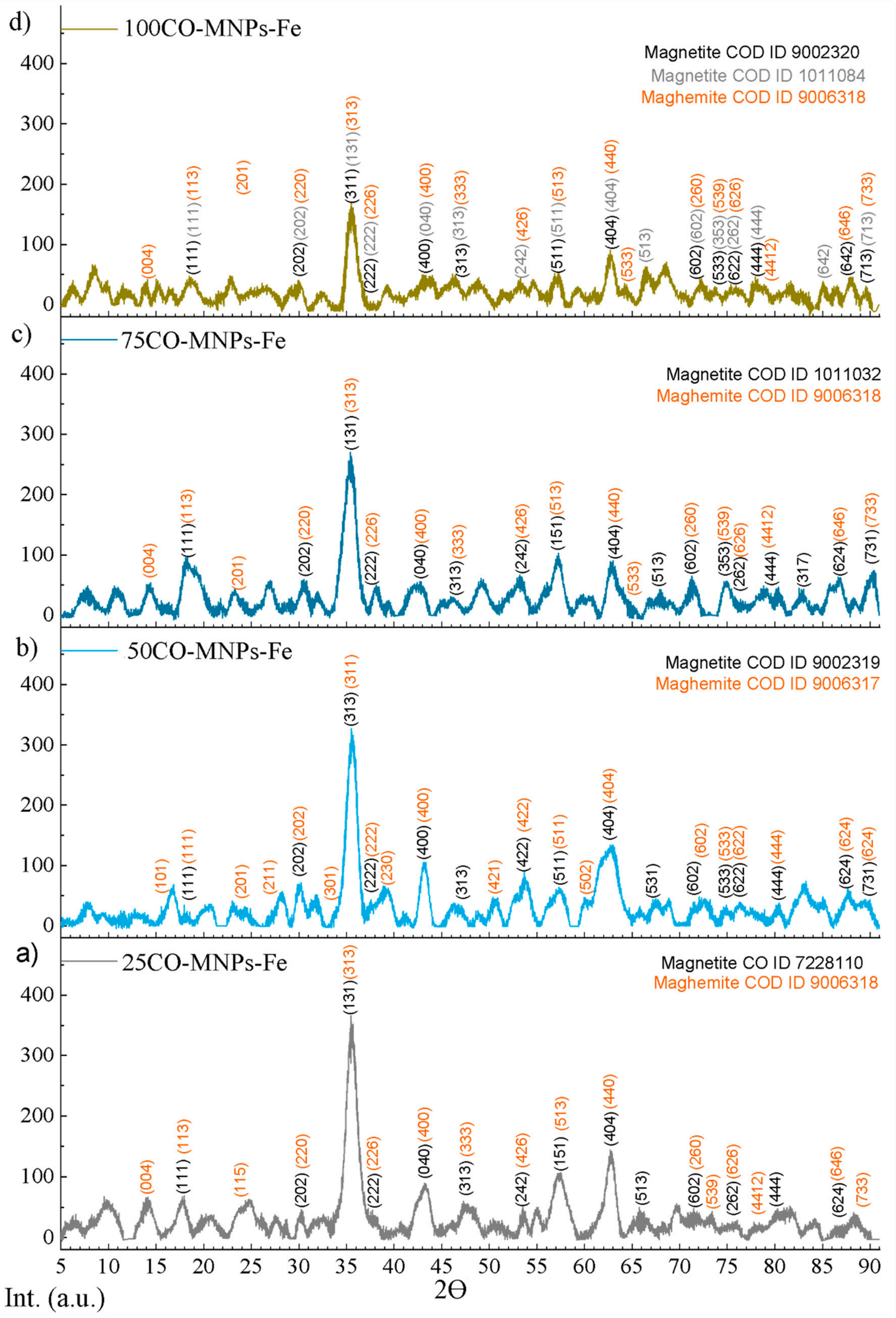

Figure S3c. Indexed XRD patterns of the samples: a) 25CO-MNPs-Fe, b) 50CO-MNPs-Fe, c) 75CO-MNPs-Fe and d) 100CO-MNPs-Fe.

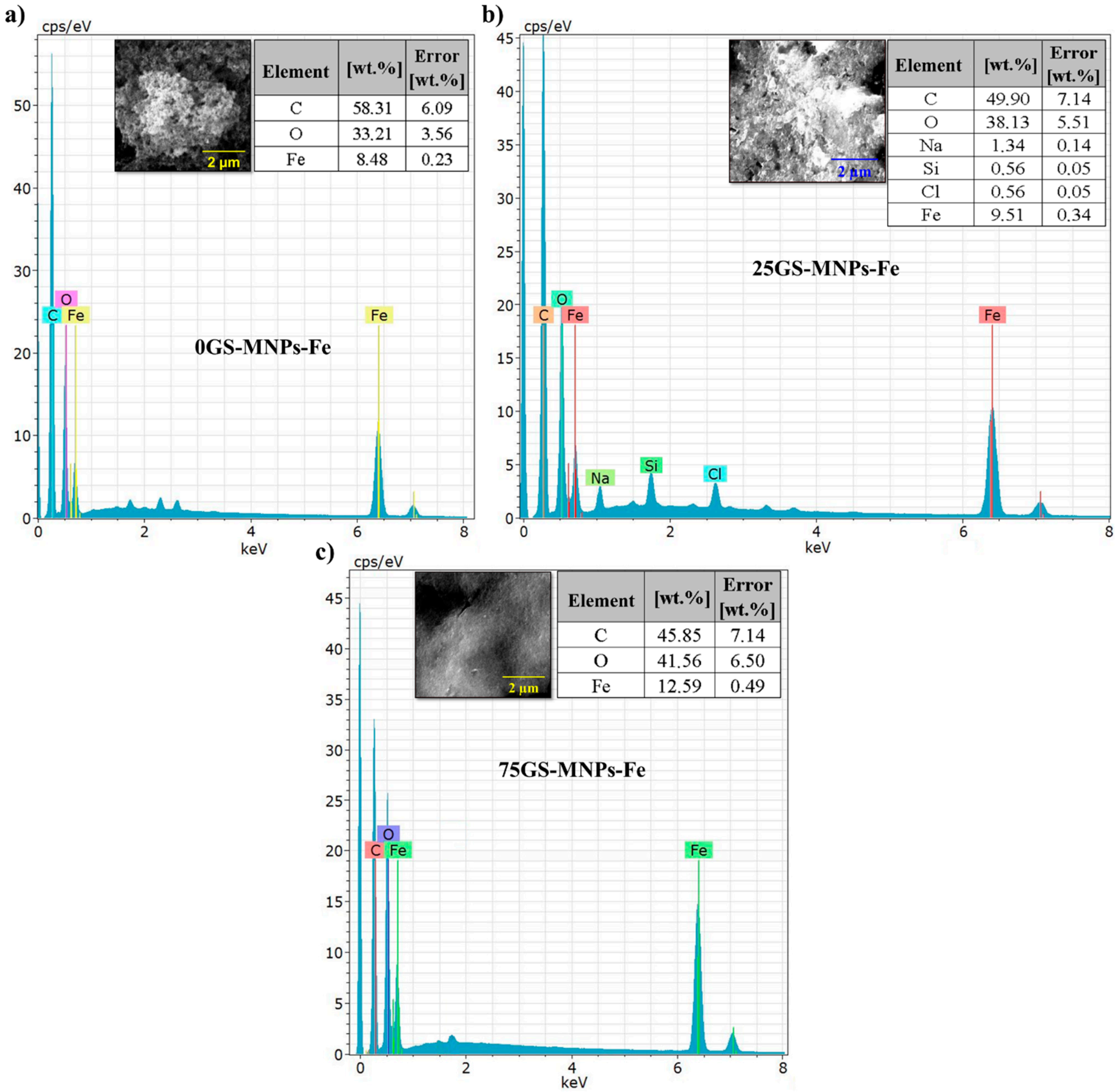

Element	[wt.%]	Error [wt.%]
C	58.31	6.09
O	33.21	3.56
Fe	8.48	0.23

Element	[wt.%]	Error [wt.%]
C	49.90	7.14
O	38.13	5.51
Na	1.34	0.14
Si	0.56	0.05
Cl	0.56	0.05
Fe	9.51	0.34

Element	[wt.%]	Error [wt.%]
C	45.85	7.14
O	41.56	6.50
Fe	12.59	0.49

Figure S4. EDS data of the samples a) 0GS-MNPs-Fe, b) 25GS-MNPs-Fe, and c) 75GS-MNPs-Fe.

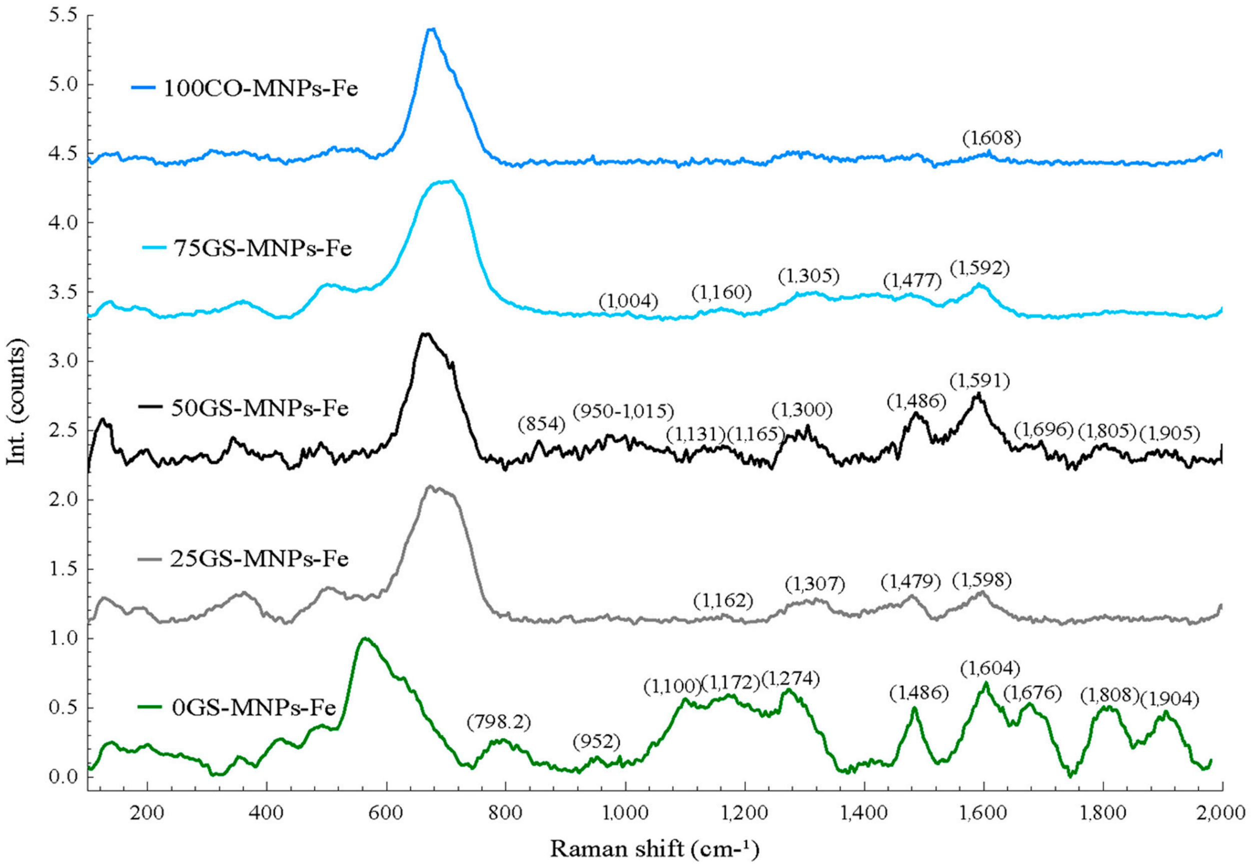

Figure S5. Full Raman spectra (100-2000 cm^{-1}) of the samples: 100CO-MNPs-Fe, 75GS-MNPs-Fe, 50GS-MNPs-Fe, 25GS-MNPs-Fe and 0GS-MNPs-Fe.

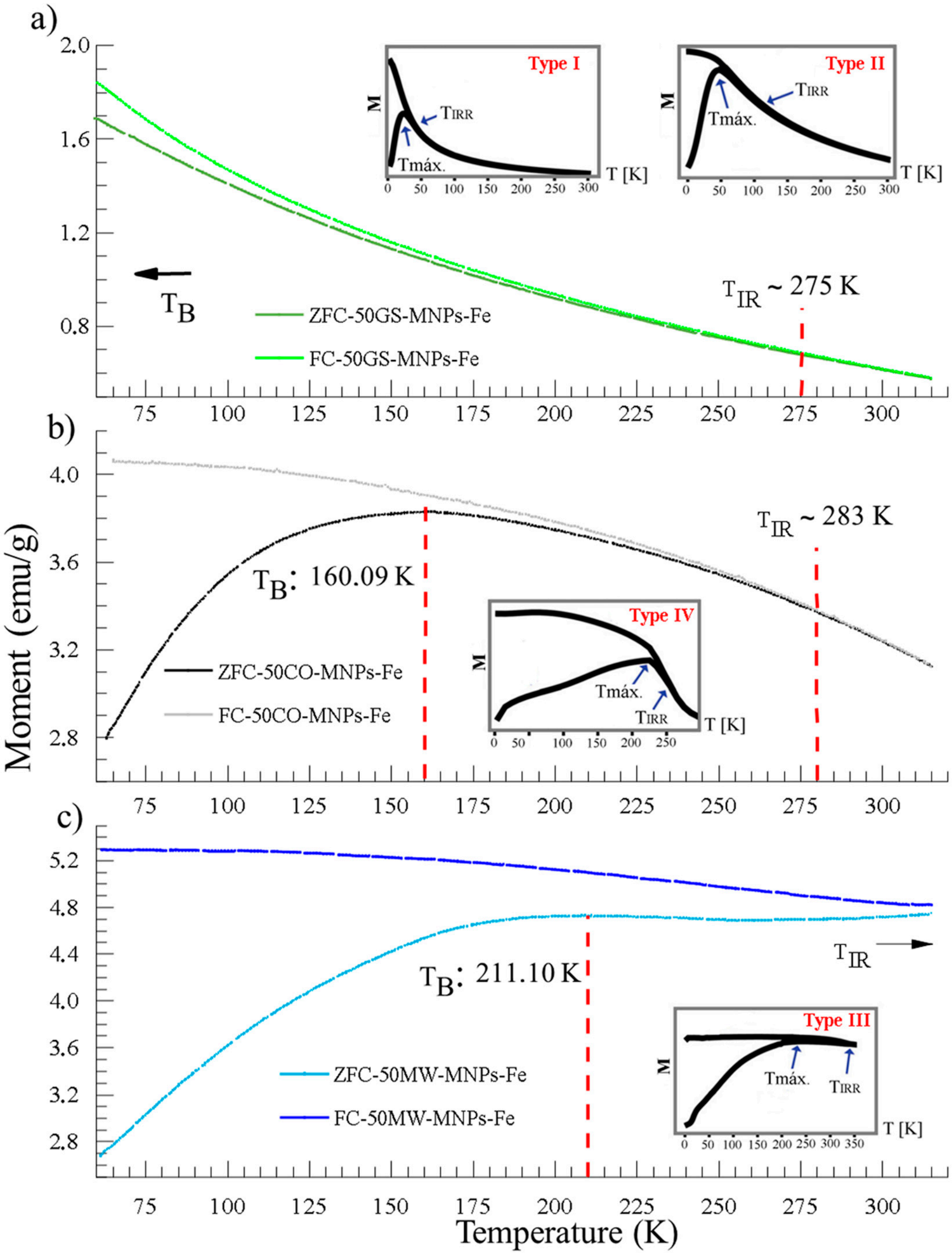

Figure S6. ZFC/FC curve of the samples a) 50GS-MNPs-Fe, b) 50CO-MNPs-Fe and c) 50MW-MNPs-Fe. The types of curves (I, II, III, IV) were assigned according to (1).

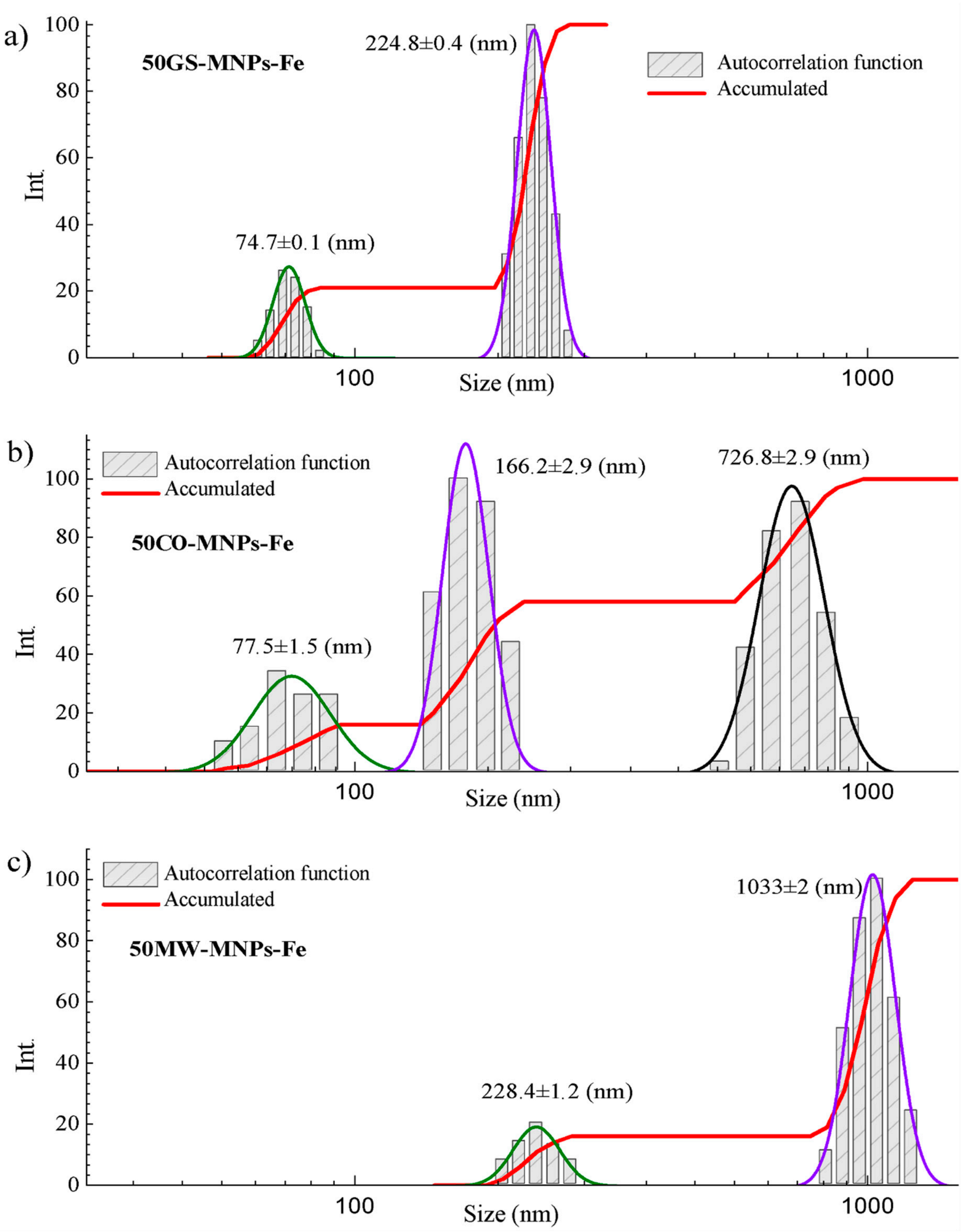

Figure S7. Size distribution of the MNPs-Fe corresponding to the samples; a) 50GS-MNPs-Fe, b) 50CO-MNPs-Fe and c) 50MW-MNPs-Fe.

Supplementary Tables

Table S1. Produced weight of MNPs-Fe obtained by GS, MW and CO

Samples	Average weight (mg)
0GS-MNPs-Fe	8.97 ± 2.32
10GS-MNPs-Fe	13.43 ± 5.26
25GS-MNPs-Fe	25.93 ± 6.23
50GS-MNPs-Fe	88.13 ± 13.33
75GS-MNPs-Fe	95.03 ± 9.85
100GS-MNPs-Fe	84.20 ± 3.10
10CO-MNPs-Fe	79.63 ± 6.20
25CO-MNPs-Fe	79.73 ± 5.61
50CO-MNPs-Fe	72.33 ± 8.96
75CO-MNPs-Fe	68.67 ± 5.70
100CO-MNPs-Fe	64.73 ± 8.90
10MW-MNPs-Fe	79.30 ± 3.87
25MW-MNPs-Fe	79.97 ± 6.85
50MW-MNPs-Fe	80.10 ± 4.61
75MW-MNPs-Fe	85.87 ± 5.56

Table S2. Average roughness, obtained from the AFM topographies, for the samples obtained by GS.

Average roughness - Ra (pm)					
ID	0GS-MNPs-Fe	25GS-MNPs-Fe	50GS-MNPs-Fe	75GS-MNPs-Fe	100GS-MNPs-Fe
1	153	186	122	139	81
2	74	181	78	70	42
3	118	105	61	116	69
4	93	107	63	50	53
5	110	66	90	56	59
6	75	131	102	94	42
7	73	119	51	61	62
8	86	143	102	57	61
9	117	183	71	51	46
10	68	132	45	67	47
11	62	113	96	71	50
12	172	97	103	86	66
13	105	115	66	84	48
14	166	75	74	52	47
15	78	85	64	63	63
16	183	146	77	61	54
17	86	117	54	92	69
18	65	132	56	52	49
19	101	108	72	65	74
20	78	134	112	99	75
**Average**	103	124	78	74	58
**SD**	±38	±33	±22	±24	±12

Table S3a. Active Raman bands of functional groups found in the Raman spectra of the 0GS-MNPs-Fe, 25GS-MNPs-Fe, 50GS-MNPs-Fe, 75GS-MNPs-Fe and 100CO-MNPs-Fe samples.

Active Raman bands of functional groups (cm^{-1}) - HORIBA	Raman bands in samples (cm^{-1})				
	0GS-MNPs-Fe	25GS-MNPs-Fe	50GS-MNPs-Fe	75GS-MNPs-Fe	100CO-MNPs-Fe
550-800, δ(C-Cl) (strong)	564.6 (strong)	-	-	-	-
600-1300, ν(C-C) aliphatic (medium)<br>630-790, ν(C-S) aliphatic (strong)<br>800-970, ν(O-C-O) (medium)	628.4 (strong)	-	-	-	-
	645.7 (strong)	-	710 (strong)	-	-
	798.2 (medium)	-	854 (medium)	-	-
600-1300, ν(C-C) aliphatic (medium)<br>800-970, ν(O-C-O) (medium)<br>1000, ν(C-C) aromatic (medium)	952 (medium)	-	950-1,015 (medium)	1,004 (weak)	-
600-1300, ν(C-C) aliphatic (medium)<br>1080-1100, ν(C-S) aromatic (strong)	1,100 (strong)	-	1,131 (medium)	-	-
600-1300, ν(C-C) aliphatic (medium)<br>1060-1150, ν_{as}(C-O-C) (weak)<br>1000-1250, ν(C=S) (strong)	1,172 (strong)	1,162 (weak)	1,165 (medium)	1,160 (weak)	-
600-1300, ν(C-C) aliphatic (medium)<br>1000-1250, ν(C=S) (strong)	1,274 (strong)	1,307 (medium)	1,300 (medium)	1,305 (media)	-
1400-1470, δ_{as}(CH_3), δ(CH_2) (medium)<br>1450,1500, ν(C-C) aromatic (medium)	1,486 (medium)	1,479 (medium)	1,486 (medium)	1,477 (medium)	-
~1600, δ(H_2O) (weak wide)<br>1500-1900, ν(C=C) (strong)	1,604 (strong)	1,598 (medium)	1,591 (strong)	1,592 (medium)	1,608 (weak)
1500-1900, ν(C=C) (strong)<br>1680-1820, ν(C=O) (medium)	1676 (strong)	-	1696 (medium)	-	-
1500-1900, ν(C=C) (strong)<br>1680-1820, ν(C=O) (medium)	1,808 (strong)	-	1,805 (medium)	-	-
1500-1900, ν(C=C) (strong)	1,904 (strong)	-	1,905 (medium)	-	-
Stretching symmetrical (ν), asymmetric (ν_{as}), symmetrical bending (δ), asymmetric (δ_{as})					

Table S3b. Active bands found in the samples 0GS-MNPs-Fe, 25GS-MNPs-Fe, 50GS-MNPs-Fe, 75GS-MNPs-Fe and 100CO-MNPs-Fe by FT-IR

FT-IR Bands, Functional Group	Bands obtained in the FT-IR spectra / sample				
	0GS-MNPs-Fe	25GS-MNPs-Fe	50GS-MNPs-Fe	75GS-MNPs-Fe	100CO-MNPs-Fe
(3700-3584)ν (O-H), alcohols	3,866 (weak)	3,886 (weak)	3,800 (weak)	---	---
(3700-3584)ν (O-H), alcohols	3,662 (weak)	3,706 (weak)	3,700 (weak)	3,690 (weak)	3,650 (weak)
(3000-2840),ν (C-H) alkanes (CH_3)<br>(~2872), ν (C-H), (CH_3)<br>(~2853), ν (C-H), (CH_2)	---	---	2,856 (weak)	2,862 (weak)	---
(2600-2550), ν (S-H), compounds S	2,576 (weak)	2,586 (weak)	2,532 (medium)	---	---
(2700-2250), ν (N-H), amine salts	2,336 (weak)	2,334 (weak)	2,350 (medium)	2,348 (weak)	2,354 (weak)
(2000-1900), ν (C=C=C), alkenes<br>(1870-1540), ν (C=O) ketones<br>(~ 1640), δ (H_2O)	1,960 (weak)	1,932 (weak)	1,886 (medium)	---	1,776 (weak)
(1670-1600), ν (C=C), (lineal, cíclicos)<br>(1600-1585), δ (C-C), (aromatic)<br>(1870-1540), ν (C=O), ketones<br>(1710-1685), ν (C=O), aldehydes<br>(~ 1640), δ (H_2O)	1,698 (medium)	1,580 (medium)	1,600 (medium)	1,638 (weak)	---
(1350-1150), ω, τ (C-H), (CH_2)<br>(1300-1000), δ (C-H), (aromatic)<br>(1260-1000), ν (C-O), alcohols<br>(1300-1000), ν(C-C-C), δ(C-C(=O)), ketones<br>(1070-1030), ν(S=O), compounds S<br>(1096-1089), ν(C-Cl), halogens	1,180 (medium)	1,156 (medium)	1,120 (medium)	1,158 (medium)	---
(850-550), ν(C-Cl), halogens<br>(~850), ω(=CH_2), alkenes<br>(900-675), δ(C-H), aromatic	870 (medium)	868 (medium)	822 (medium)	828 (medium)	---
(850-550), ν(C-Cl), halógens<br>(700-450), ν (Fe-O)	578	546	640	578	---
(700-450), ν (Fe-O)	508	502	536	520	550
Stretching symmetrical (ν), asymmetrical (ν_{as}), symmetrical bending (δ), asymmetrical (δ_{as}), Scissoring (δ_s), Rocking (ρ), Wagging (ω), Twisting (τ).					

Table S4. Parameters of crystalline structures obtained from XRD.

Samples	FWHW	2θ	D_c	a (A)	Iron oxide	Space Group	COD ID
0GS-MNPs-Fe	0.24	35.09	34.64	8.474954	35.3% Magnetite	Fd-3m – cubic	1011032
	---	---	---	---	64.70% Hematite	R-3c – trigonal-hexagonal	9000139
25GS-MNPs-Fe	0.25	35.08	32.40	8.478080	39.5% Magnetite	Fd-3m – cubic	10100369
	---	---	---	---	53.0 % Maghemite	4_32_12 – tetragonal	9006318
	---	---	---	---	7.5% Hematite	C2/c – monoclinic	2108027
50GS-MNPs-Fe	0.75	35.38	10.97	8.406839	47.0% Magnetite	Fd-3m – cubic	1011032
	---	---	---	---	53.0% Maghemite	Fd-3m – cubic	9006316
75GS-MNPs-Fe	0.47	35.37	17.43	8.405744	47.0% Magnetite	Fd-3m – cubic	1011032
	---	---	---	---	53.0% Maghemite	P 4_132 – cubic	9006317
100GS-MNPs-Fe	0.87	35.74	9.53	8.331638	6.6% Magnetite	Fd-3m – cubic	1011084
	---	---	---	---	94.3% Maghemite	$P4_32_12$ – tetragonal<br>$P4_12_12$ – tetragonal	9006318<br>1528612
25CO-MNPs-Fe	1.18	35.39	6.97	8.405071	88.0% Magnetite	Fd-3m – cubic	7228110
	---	---	---	---	12.0% Maghemite	$P4_32_12$ – tetragonal	9006318
50CO-MNPs-Fe	1.41	35.35	5.87	8.415581	63.5% Magnetite	Fd-3m (227) – cubic	9002319
	---	---	---	---	36.5% Maghemite	$P4_132$ – cubic	9006317
75CO-MNPs-Fe	1.72	35.24	4.78	8,440935	8.9% Magnetite	Fd-3m – cubic	1011032
	---	---	---	---	91.1% Maghemite	$P4_32_12$ – tetragonal	9006318
100CO-MNPs-Fe	1.03	35.41	7.99	8.400700	98.0% Magnetite	Fd-3m – cubic	9002320<br>1011084
	---	---	---	---	2.0% Maghemite	$P4_32_12$ – tetragonal	9006318
25MW-MNPs-Fe	0.70	35.33	11.71	8.420069	34,1% Magnetite	Fd-3m – cubic	7228110
	---	---	---	---	65.9% Maghemite	$P4_12_12$ – tetragonal	1528612
50MW-MNPs-Fe	0.80	35.25	10.24	8.436802	91.2% Magnetite	Fd-3m – cubic	9005842
	---	---	---	---	8.8% Maghemite	$P4_132$ – cubic	1528612
75MW-MNPs-Fe	1.06	35.33	7.74	8.418517	10.0% Magnetite	Fd-3m – cubic	9005814
	---	---	---	---	90.0% Maghemite	$P4_12_12$ – tetragonal<br>$P4_32_12$ – tetragonal	1528612<br>9012692

Table S5a. Log_{10} (CFU) count of antibacterial PTT assay groups for *S. aureus* and *E. coli.*

S. aureus								
Class # (Tukey Test)	1	2	3	4	5	6	7	9
Colony Forming Units	*S. aureus*	*S. aureus* + Light	*S. aureus* + 50GS-MNPs-Fe	*S. aureus* + 50GS-MNPs-Fe + Light	*S. aureus* + 50CO-MNPs-Fe	*S. aureus* + 50CO-MNPs-Fe + Light	*S. aureus* + 50MW-MNPs-Fe	*S. aureus* + 50MW-MNPs-Fe + Light
Log_{10} (CFU/mL)	5.142	5.204	4.931	0.000	5.069	0.000	5.107	0.000
Log_{10} (CFU/mL)	5.089	5.158	4.873	0.000	5.158	0.000	5.069	3.301
Log_{10} (CFU/mL)	5.049	5.174	4.727	0.000	5.125	0.000	5.049	0.000
Average Log_{10}(CFU/mL)	5.093	5.179	4.844	0.000	5.118	0.000	5.075	1.100
Desv. St.	0.047	0.023	0.105	0.000	0.045	0.000	0.029	1.906
E. coli								
Class # (Tukey Test)	1	2	3	4	5	6	7	9
Colony Forming Units	*E. coli*	*E. coli* + Light	*E. coli* + 50GS-MNPs-Fe	*E. coli* + 50GS-MNPs-Fe + Light	*E. coli* + 50CO-MNPs-Fe	*E. coli* + 50CO-MNPs-Fe + Light	*E. coli* + 50MW-MNPs-Fe	*E. coli* + 50MW-MNPs-Fe + Light
Log_{10} (CFU/mL)	5.0280	5.2041	5.3802	4.1072	5.1249	0.0000	4.9823	0.0000
Log_{10} (CFU/mL)	5.1742	5.2711	5.3705	3.4472	5.1072	0.0000	5.0887	0.0000
Log_{10} (CFU/mL)	4.9574	5.1742	5.4346	0.0000	5.2041	2.6021	5.2585	0.0000
Average Log_{10}(CFU/mL)	5.0532	5.2164	5.3951	2.5181	5.1454	0.8674	5.1098	0.0000
Desv. St.	0.1105	0.0496	0.0345	2.2056	0.0516	1.5023	0.1393	0.0000

Table S5b. Means Comparisons for *S. aureus.*

Tukey Test - *S. aureus* groups						
Class #	Mean Diff.	SEM	q value	Prob.	Alpha	Sig
Class2 Class1	0.08557	0.55143	0.21947	1	0.001	0
Class3 Class1	-0.24956	0.55143	0.64002	0.99974	0.001	0
Class3 Class2	-0.33513	0.55143	0.85949	0.99823	0.001	0
Class4 Class1	-5.09331	0.55143	13.0624	1.77889E-6	0.001	1
Class4 Class2	-5.17888	0.55143	13.28187	1.40295E-6	0.001	1
Class4 Class3	-4.84375	0.55143	12.42238	3.5664E-6	0.001	1
Class5 Class1	0.02427	0.55143	0.06224	1	0.001	0
Class5 Class2	-0.06131	0.55143	0.15723	1	0.001	0
Class5 Class3	0.027383	0.55143	0.70226	0.99951	0.001	0
Class5 Class4	5.11757	0.55143	13.12464	1.66305E-6	0.001	1
Class6 Class1	-5.09331	0.55143	13.0624	1.77889E-6	0.001	1
Class6 Class2	-5.17888	0.55143	13.28187	1.40295E-6	0.001	1
Class6 Class3	-4.84375	0.55143	12.42238	3.5664E-6	0.001	1
Class6 Class4	0	0.55143	0	1	0.001	0
Class6 Class5	-5.11757	0.55143	13.12464	1.66305E-6	0.001	1
Class7 Class1	-0.01802	0.55143	0.04622	1	0.001	0
Class7 Class2	-0.1036	0.55143	0.26569	1	0.001	0
Class7 Class3	0.23154	0.55143	0.5938	0.99984	0.001	0
Class7 Class4	5.07528	0.55143	13.01618	1.87014E-6	0.001	1
Class7 Class5	-0.04229	0.55143	0.10846	1	0.001	0
Class7 Class6	5.07528	0.55143	13.01618	1.87014E-6	0.001	1
Class8 Class1	-3.99296	0.55143	10.24044	4.30556E-5	0.001	1
Class8 Class2	-4.07854	0.55143	10.4599	3.31411E-5	0.001	1
Class8 Class3	-3.7434	0.55143	9.60042	9.38762E-5	0.001	1
Class8 Class4	1.10034	0.55143	282196	0.51335	0.001	0
Class8 Class5	-4.01723	0.55143	10.30268	3.99643E-5	0.001	1
Class8 Class6	1.10034	0.55143	2.82196	0.51335	0.001	0
Class8 Class7	-3.97494	0.55143	10.19422	4.55116E-5	0.001	1

Sig. equals 1indicates that the difference of the means is significant at the 0.001 level.

Sig. equals 0 indicates that the difference of the means is not significant at the 0.001 level.

Table S5c. Means Comparisons for *E. coli.*

Tukey Test - *E. coli* groups						
Class #	Mean Diff.	SEM	q value	Prob.	Alpha	Sig
Class2 Class1	0.16324	0.77241	0.29887	1	0.001	0
Class3 Class1	0.34187	0.77241	0.62592	0.99974	0.001	0
Class3 Class2	0.17863	0.77241	0.32705	1	0.001	0
Class4 Class1	-2.53509	0.77241	4.6415	0.06981	0.001	0
Class4 Class2	-2.69833	0.77241	4.94037	0.04716	0.001	0
Class4 Class3	-2.87695	0.77241	5.26742	0.03041	0.001	0
Class5 Class1	0.09221	0.77241	0.16883	1	0.001	0
Class5 Class2	-0.07102	0.77241	0.13004	1	0.001	0
Class5 Class3	-0.24965	0.77241	0.45709	0.99995	0.001	0
Class5 Class4	2.6273	0.77241	4.81033	0.056	0.001	0
Class6 Class1	-4.18586	0.77241	7.6639	0.00115	0.001	0
Class6 Class2	-4.34909	0.77241	7.96277	7.68451E-4	0.001	1
Class6 Class3	-4.52772	0.77241	8.28982	4.98996E-4	0.001	1
Class6 Class4	-1.65077	0.77241	3.0224	0.43366	0.001	0
Class6 Class5	-4.27807	0.77241	7.83273	9.13727E-4	0.001	1
Class7 Class1	0.05661	0.77241	0.10365	1	0.001	0
Class7 Class2	-0.10662	0.77241	0.19522	1	0.001	0
Class7 Class3	-0.28525	0.77241	0.52227	0.99993	0.001	0
Class7 Class4	2.5917	0.77241	4.74515	0.061	0.001	0
Class7 Class5	-0.0356	0.77241	0.06518	1	0.001	0
Class7 Class6	4.24247	0.77241	7.76755	9.96869E-4	0.001	1
Class8 Class1	-5.05321	0.77241	9.25194	1.44916E-4	0.001	1
Class8 Class2	-5.21645	0.77241	9.55081	9.98233E-5	0.001	1
Class8 Class3	-5.39508	0.77241	9.87786	6.67556E-5	0.001	1
Class8 Class4	-2.51812	0.77241	4.61044	0.07267	0.001	0
Class8 Class5	-5.14542	0.77241	9.42077	1.17342E-4	0.001	1
Class8 Class6	-0.86735	0.77241	1.58804	0.94207	0.001	0
Class8 Class7	-5.10983	0.77241	9.35559	1.27262E-4	0.001	1

Sig. equals 1indicates that the difference of the means is significant at the 0.001 level.

Sig. equals 0 indicates that the difference of the means is not significant at the 0.001 level.

Table S6a. Log (inhibitor) vs. response - Variable slope (four parameters) - 24 h, with animal cell line ATCC RAW 264.7.

Log(inhibitor) vs. response - Variable slope (four parameters)			
Best-fit values	50GS-MNPs-Fe	50CO-MNPs-Fe	50MW-MNPs-Fe
Bottom	54.11	57.2	33.77
Top	97.3	96.63	99.82
LogIC50	2.503	2.779	3.105
HillSlope	-3.972	-2.644	-1.794
IC50	318.2	601.6	1274
Span	43.2	39.43	66.05
95% CI (profile likelihood)	50GS-MNPs-Fe	50CO-MNPs-Fe	50MW-MNPs-Fe
Bottom	47.62 to 59.31	42.14 to 63.49	-323.6 to 51.33
Top	94.26 to 100,7	94.48 to 98.93	98.14 to 101.6
LogIC50	2.412 to 2.593	2.675 to 2.982	2.933 to 4.134
HillSlope	-infinity to -1.787	-6.820 to -1.397	-2.665 to -1.057
IC50	258.3 to 391.4	473.2 to 958.5	857.0 to 13619
Goodness of Fit	50GS-MNPs-Fe	50CO-MNPs-Fe	50MW-MNPs-Fe
Degrees of Freedom	26	26	26
R squared	0.9152	0.9189	0.9558
Sum of Squares	933.4	494.4	295
Sy.x	5.992	4.361	3.368
Number of points	50GS-MNPs-Fe	50CO-MNPs-Fe	50MW-MNPs-Fe
# X values	30	30	30
# Y values	30	30	30

Table S6b. Log (inhibitor) vs. response - Variable slope (four parameters) – 8 days, with animal cell line ATCC RAW 264.7.

Log(inhibitor) vs. response -- Variable slope (four parameters)			
Best-fit values	50MW-MNPs-Fe	50CO-MNPs-Fe	50GS-MNPs-Fe
Bottom	23.41	20.33	28.95
Top	97.23	100.2	100.1
LogIC50	2.338	2.716	2.664
HillSlope	-1.847	-0.8897	-2.407
IC50	217.7	520.2	461.4
Span	73.82	79.91	71.13
95% CI (profile likelihood)	50MW-MNPs-Fe	50CO-MNPs-Fe	50GS-MNPs-Fe
Bottom	13.39 to 30.56	to 45.55	14.48 to 37.46
Top	93.04 to 101.8	92.63 to +infinity	96.69 to 103.6
LogIC50	2.231 to 2.460	2.305	2.572 to 2.794
HillSlope	-2.638 to -1.273	-2.009 to 0.1726	-3.912 to -1.537
IC50	170.4 to 288.6	201.9	373.5 to 621.8
Goodness of Fit	50MW-MNPs-Fe	50CO-MNPs-Fe	50GS-MNPs-Fe
Degrees of Freedom	26	26	26
R squared	0.9542	0.8633	0.948
Sum of Squares	1222	2058	1117
Sy.x	6.855	8.898	6.555
Number of points	50MW-MNPs-Fe	50CO-MNPs-Fe	50GS-MNPs-Fe
# X values	30	30	30
# Y values	30	30	30

References:

1. Sharma SK. Complex magnetic nanostructures: Synthesis, assembly and applications. Complex Magnetic Nanostructures: Synthesis, Assembly and Applications. 2017. 1–464 p.
